# Effects of lasalocid, narasin, or virginiamycin supplementation on rumen parameters and performance of beef cattle fed forage-based diet

**DOI:** 10.1093/jas/skad108

**Published:** 2023-04-12

**Authors:** Alexandre Arantes Miszura, Rodrigo S Marques, Daniel Montanher Polizel, Bruno Ieda Cappellozza, Vinicius Alves Cruz, Makayla Anne Ogg, José Paulo Roman Barroso, Gabriela Bagio Oliveira, André Storti Martins, Arnaldo Cintra Limede, Evandro Maia Ferreira, Vinícius N Gouvêa, Alexandre Vaz Pires

**Affiliations:** Department of Animal Nutrition and Animal Production, University of São Paulo, Pirassununga 13635-000, Brazil; Department of Animal and Range Science, Montana State University, Bozeman, MT 59717, USA; Department of Animal Science, “Luiz de Queiroz” College of Agriculture, University of São Paulo, Piracicaba 13418-900, Brazil; Chr. Hansen, Hørsholm 2970, Denmark; Department of Animal and Range Science, Montana State University, Bozeman, MT 59717, USA; Department of Animal and Range Science, Montana State University, Bozeman, MT 59717, USA; Department of Animal Nutrition and Animal Production, University of São Paulo, Pirassununga 13635-000, Brazil; Department of Animal Nutrition and Animal Production, University of São Paulo, Pirassununga 13635-000, Brazil; Department of Animal Nutrition and Animal Production, University of São Paulo, Pirassununga 13635-000, Brazil; Department of Animal Nutrition and Animal Production, University of São Paulo, Pirassununga 13635-000, Brazil; Department of Animal Science, “Luiz de Queiroz” College of Agriculture, University of São Paulo, Piracicaba 13418-900, Brazil; Texas A&M AgriLife Research and Extension Center, Amarillo, TX 79106, USA; Department of Animal Science, Texas A&M University, College Station, TX 77845, USA; Department of Animal Science, “Luiz de Queiroz” College of Agriculture, University of São Paulo, Piracicaba 13418-900, Brazil

**Keywords:** *Bos indicus*, digestibility, feed additives, forage, ionophore, performance, ruminal parameters

## Abstract

Two experiments were designed to evaluate the impacts of supplementing lasalocid (**LAS**), narasin (**NAR**), or virginiamycin (**VRM**) on rumen fermentation parameters, apparent nutrient digestibility, and blood parameters (Exp. 1), as well as feed intake and performance (Exp. 2) of Nellore cattle consuming a forage-based diet. In Exp. 1, 32 rumen-fistulated Nellore steers (initial shrunk body weight [**BW**] = 355 ± 4.4 kg) were assigned to a randomized complete block design. Within block, animals were randomly assigned to one of four treatments: 1) forage-based diet without feed additives (**CON**), 2) CON diet plus 13 mg/kg of dry matter (**DM**) of NAR, 3) CON diet plus 20 mg/kg of DM of sodium LAS, or 4) CON diet plus 20 mg/kg of DM of VRM. No treatment effects were detected (*P* ≥ 0.32) for intake and apparent digestibility of nutrients. Steers fed NAR had the lowest (*P* ≤ 0.01) molar proportion of acetate on day 28, 56, and 112 vs. CON, LAS, and VRM steers, whereas acetate did not differ (*P* ≥ 0.25) between LAS, VRM, and CON steers from day 28 to 84. On day 112, steers fed LAS had a lower (*P* < 0.02) molar proportion of acetate vs. VRM and CON, whereas it did not differ between CON and VRM (*P* > 0.33). Steers receiving NAR had a greater (*P* ≤ 0.04) ruminal propionate vs. CON, LAS, and VRM, whereas LAS steers had greater (*P* < 0.04) propionate vs. CON and VRM steers on day 28 and 112, and it did not differ (*P* > 0.22) between CON and VRM. In Exp. 2, 160 Nellore bulls were blocked by initial shrunk BW (212 ± 3.1 kg) in a 140-d feedlot trial. Diets contained the same treatments used in Exp. 1. Bulls fed NAR had greater (*P* < 0.02) average daily gain (**ADG**) vs. CON and VRM, and similar (*P* = 0.17) ADG between NAR and LAS, whereas ADG did not differ (*P* > 0.28) between LAS, VRM, and CON bulls. A treatment effect was detected (*P* = 0.03) for dry matter intake, being greater in NAR vs. CON, LAS, and VRM bulls, and similar (*P* > 0.48) between CON, LAS, and VRM bulls. A tendency was detected (*P* = 0.09) for feed efficiency, which was greater (*P* < 0.02) in NAR bulls vs. CON and VRM, and similar (*P* = 0.36) between NAR and LAS bulls. From day 112 to 140, bulls receiving NAR were heavier (*P* < 0.03) vs. CON, LAS, and VRM bulls, but no differences were observed (*P* > 0.51) between CON, LAS, and VRM bulls. Collectively, ruminal fermentation profile and intake were impacted by NAR supplementation, which partially contributed to the enhanced performance of Nellore bulls receiving a forage-based diet.

## Teaser

Supplementing narasin to beef cattle for 140 d might be a feasible nutrition tool to optimize rumen fermentation characteristics and productivity in grazing cattle systems.

## Introduction

Dietary feed additives can promote better dietary digestibility and nutrient utilization, alter ruminal fermentative routes, and improve cattle growth and efficiency (Tedeschi et al., 2003), resulting in enhanced productivity and profitability in beef cattle systems. Nevertheless, the majority of research conducted to date using dietary feed additives has focused on high-concentrate diets (Tedeschi et al., 2003; Duffield et al., 2012; Ellis et al., 2012). A limited number of studies have investigated the influence of feed additives on *Bos indicus* Nellore cattle fed forage-based diets. Additionally, there is a significant gap to be filled by research involving molecules capable of altering the fermentation process and, consequently, performance of Nellore cattle fed forage-based diets (Fieser et al., 2007; Beck et al., 2014).

Several ionophores (lasalocid [**LAS**], monensin, salinomycin, laidlomycin, and narasin [**NAR**]) are commercially available and commonly fed in many phases of beef production systems. Although their ruminal mechanisms are similar, performance might vary depending on dosage, animal, and diet (Nagaraja et al., 1987; Tedeschi et al., 2003; Bretschneider et al., 2008). For instance, NAR is an ionophore produced by *Streptomyces aureofaciens*, which positively affects ruminal fermentation and performance of beef cattle consuming forage-based diets (Silva et al., 2015; Polizel et al., 2018; Limede et al., 2021). LAS is another ionophore derived from strains of *Streptomyces lasaliensis*, which also modifies rumen environment and favorably enhances productivity of beef cattle consuming forage-based diets (Golder and Lean, 2016). Virginiamycin (**VRM**) is a nonionophore produced by a specific strain of *Streptomyces virginiae* (De Somer and VanDijck, 1955), which has been utilized in beef cattle production systems to modulate feedlot cattle rumen pH (Godfrey et al., 1995), reducing the risk of acidosis (Rogers et al., 1995). Additionally, VRM has been shown to enhance ruminal propionate, performance, and decrease liver abscess incidence in beef cattle (Rogers et al., 1995; Coe et al., 1999; Salinas-Chavira et al., 2009).

Thus, we hypothesized that supplementing or not LAS, NAR, or VRM would affect nutrient digestibility, change rumen fermentation parameters, and increase productivity of *B. indicus* Nellore cattle fed a forage-based diet. Therefore, the objective of this experiment was to evaluate the impacts of supplementing NAR, LAS, or VRM for 140 d on rumen fermentation parameters, apparent nutrient digestibility, and blood parameters (Exp. 1), as well as feed intake and growth performance (Exp. 2) of *B. indicus* Nellore cattle consuming forage-based diet.

## Materials and Methods

This study was conducted at the University of São Paulo, Piracicaba campus (USP/ESALQ; Piracicaba, SP, Brazil; 22°43ʹ31″S, 47°38ʹ51″W, and 524 m elevation). Experimental procedures involving animals were reviewed and approved by the Ethics Committee on Use of Animals of School of Veterinary Medicine and Animal Science (University of Sao Paulo; CEUA/FMVZ; protocol #7491171017). The experimental design, measurements, and analyses used herein are similar described by Limede et al. (2021).

### Experiment 1—Rumen fermentation parameters

#### Animals, housing, and treatments

Thirty-two rumen-fistulated Nellore (*B. indicus*) steers (initial shrunk body weight [**BW**] = 355 ± 4.4 kg; age = 24 ± 1.0 mo) were assigned to individual pens (concrete-surface; 2 × 2 m, with a feed bunk and waterer) in a randomized complete block design (*n* = 8), according to their initial shrunk BW. Within block (*n* = 8), animals were randomly assigned to one of four treatments: 1) forage-based diet without feed additives (**CON**; *n* = 8), 2) CON diet plus 13 mg/kg of dry matter (**DM**) of NAR (Zimprova; Elanco Animal Health, São Paulo, SP, Brazil; *n* = 8), 3) CON diet plus 20 mg/kg of DM of sodium LAS (Taurotec; Zoetis, São Paulo, SP, Brazil; *n* = 8), or 4) CON diet plus 20 mg/kg of DM of VRM (V-Max; Phibro Animal Health Corporation, Guarulhos, SP, Brazil; *n* = 8). The administration rates of NAR, LAS, and VRM used herein were according to the manufacturer’s recommendation. The experimental period lasted 140 d and was divided into five periods of 28 d each (0, 28, 56, 84, 112, and 140 d) as in Limede et al. (2021).

From day 0 to 140, steers were fed daily with 99% of chopped coastcross haylage [*Cynodon dactylon* (L.) Pers] and 1% of concentrate (50% ground corn and 50% ground soybean hulls; as-fed basis), which was used as a delivery vehicle for the additives (Table 1). Haylage was chopped using a vertical mixer (Mixer VM8B, DeLaval International AB, Tumba, Sweden) for about 15 min, which resulted in the following average particle size distribution: 53.3% ± 3.5% > 19 mm; 21.9% ± 1.7% > 8 mm; 12.4% ± 1.0% > 4 mm; and 12.4% ± 1.5% on bottom sieve according to Penn State Particle Separator procedures (Heinrichs, 1996; Kononoff et al., 2003). Treatments containing feed additives were separately mixed into the concentrate and offered to each steer individually. Feed additive treatment was initially included in the concentrate based on a 5.0-kg forage dry matter intake (**DMI**). For instance, for steers consuming 5.0 kg of forage, the concentrate would contain 65, 100, and 100 mg/kg of DM of NAR, LAS, and VRM for NAR, LAS, and VRM, respectively. Steers from the CON group also received the same concentrate without including feed additives. From day 0 to 140, animals were individually fed the feed additive treatments once daily (0800 h) prior to haylage feeding to avoid a small amount of concentrate being mixed with hay and compromising intake of the feed additive treatments (Limede et al., 2021). Throughout the experimental period (day 0 to 140), animals had ad libitum access to haylage (0830 h), mineral mix (offered in separate feed bunk from the haylage and treatments), and fresh water. Treatment amounts were calculated daily based on the previous day individual total forage DMI. The mineral mix (Bellmais; Trouw Nutrition, Campinas, SP, Brazil) used herein contained 152 g/kg Ca, 60 g/kg P, 40 g/kg S, 140 g/kg Na, 10 g/kg Mg, 3,750 mg/kg Zn, 1,010 mg/kg Cu, 780 mg/kg Mn, 60 mg/kg Co, 75 mg/kg I, and 19 mg/kg Se. The nutritional profile of haylage and concentrate used in the present experiment is described in Table 1.

**Table 1.** Nutritional profile of the haylage [*Cynodon dactylon* (L.) Pers] and concentrate used in Exp. 1 and 2

	Experiment 1		Experiment 2	
Item^1^	Coastcross haylage	Concentrate^2^	Coastcross haylage	Concentrate^3^
Nutrient profile, dry matter basis				
Dry matter, %	41.5	88.6	59.7	87.4
Crude protein, %	11.6	10.6	12.0	7.8
Neutral detergent fiber, %	64.6	37.2	62.0	16.9
Acid detergent fiber, %	34.4	3.5	32.4	3.8
Ash, %	9.9	3.2	6.8	4.4
Total digestible nutrients^4^, %	55.4	75.1	55.4	78.8
Net energy of maintence^5^, Mcal/kg	1.15	1.78	1.15	1.90
Net energy of gain^5^, Mcal/kg	0.59	1.15	0.59	1.26

^1^Based on nutritional profile of each ingredient, which were analyzed via wet chemistry procedures (AOAC, 1990).

^2^Concentrate: 50% ground corn and 50% ground soybean hulls (as-fed basis).

^3^Concentrate: 50% ground corn and 50% ground citrus pulp dry (as-fed basis).

^4^Calculations were performed according to the equations proposed by Weiss et al. (1992).

^5^Calculated composition using tabular values from NASEM (2016).

#### Sample collection, laboratory analyses, and measurements

Samples of haylage and concentrate were collected weekly, pooled across all weeks within each period, and analyzed for nutrient profile (Table 1). From day 23 to 27 (period 01), 51 to 55 (period 02), 79 to 83 (period 03), 107 to 111 (period 04), and 135 to 139 (period 05), total fecal production was individually collected to determine apparent nutrient digestibility analysis. Total fecal output was collected and quantified using an electronic scale (Marte AC-10K; Marte Cientifica, São Paulo, SP, Brazil) at 0800 h and 1800 h, and a representative sample (approximately 10% of wet weight) of the daily production of each steer was collected and stored at −18 °C on the same day of collection. Total tract apparent nutrient digestibility was calculated according to the formula as described by Polizel et al. (2020): TTAD (%) = ((DMI × NCDM) − (FDM × NCFM) × 100)/(DMI × NCDM), where TTAD = total tract apparent digestibility, DMI = dry matter intake, NCDM = nutrient content of the DMI (%), FDM = fecal dry matter, and NCFM = nutrient content of the fecal DM (%).

Samples of feed, orts, and feces were dried in a forced-air oven at 60 °C (AOAC, 1990; #930.15) for 96 h. Sequentially, the samples were ground through a 1-mm Wiley Mill screen (Marconi, Piracicaba, SP, Brazil). The final DM content was determined after oven-drying the samples at 105 °C for 24 h (AOAC, 1990; #934.01), and ash concentration was obtained by incinerating the samples in an oven at 550 °C for 4 h (AOAC, 1990; method #942.05). Sequential detergent fiber analyses were used to determine neutral detergent fiber (**NDF**; Van Soest et al., 1991) and acid detergent fiber (**ADF**; Goering and Van Soest, 1970) with an Ankom 2000 fiber analyzer (Ankom Tech. Corp., Macedon, NY). Sodium sulfite and heat-stable α-amylase were added to the NDF analysis. The total N was determined according to AOAC (1990; method #968.0) using the Leco TruMac N (Leco Corp., St. Joseph, MI), and the crude protein (**CP**) was obtained by multiplying the total N content by 6.25. Calculation of haylage and concentrate total digestible nutrients, net energy for maintenance and gain were performed according to Weiss et al. (1992) and the tabular values proposed by NASEM (2016).

Individual shrunk BW was collected on day 0 after 14 h of feed and water withdrawal and used to calculate initial BW and to perform the randomization into blocks and treatments. Forage, concentrate, and total DMI were recorded daily from each pen by collecting and weighing nonconsumed feed (forage only). Samples of the offered and nonconsumed feed were collected daily from each pen and dried for 24 h at 105 ± 2 °C in forced-air ovens for dry matter calculation.

On day 0 (immediately prior to the beginning of the experimental period and first treatment offer), 28, 56, 84, 112, and 140 of the experimental period at 0, 6, and 12 h after concentrate feeding, ruminal fluid samples were collected (approximately 100 mL per sample time) by squeezing the ruminal contents into four layers of cheesecloth and the ruminal fluid pH was immediately determined (Digimed-M20; Digimed Instrumentação Analítica, São Paulo, SP, Brazil). Approximately 50 mL of ruminal fluid were collected, pooled across all sampling times (0, 6, and 12 h), within each experimental period, and stored (−20 °C) for subsequent analysis of rumen ammonia and molar proportions of individual short-chain fatty acids (**SCFA**; acetate, propionate, butyrate, isobutyrate, valerate, isovalerate), as well as the acetate:propionate (**Ac:Pr**) ratio, and total SCFA. Frozen ruminal samples were prepared for analysis by thawing, centrifuging (15,000 × *g*) for 10 min at room temperature and analyzed for SCFA and rumen ammonia according to procedures described by Ferreira et al. (2016) and Broderick and Kang (1980), respectively.

Blood samples were collected on day 0 (immediately prior to the beginning of the experimental period and first treatment offer), 28, 56, 84, 112, and 140 of the experimental period at 6 h after feeding, via coccygeal venipuncture into commercial vacutainer collection tubes with glycolytic inhibitor and anticoagulant K_3_EDTA (Vacuette; Greiner Bio-One, Americana, SP, Brazil). All blood samples were immediately placed on ice, subsequently centrifuged (2,000 × *g* at 4 °C for 15 min) for plasma collection into 1.5 mL tubes (Eppendorf AG, São Paulo, SP, Brazil), and stored at −20 °C until analysis. The blood parameters were determined in the Automatic Biochemistry System—Model SBA-200 (CELM, Barueri, SP, Brazil). Commercial enzymatic kits from Labtest Diagnostic SA (Lagoa Santa, MG, Brazil) were used for the determination of plasma concentration of glucose and urea. All samples were analyzed for ruminal ammonia, glucose, and urea concentration within a single assay, and the intra-assay CV was 4.91%, 1.78%, and 4.44%, respectively.

### Experiment 2—Performance

#### Animal, housing, and experimental design

One hundred and sixty *B. indicus* Nellore yearling bulls were assigned to pens in a randomized complete block design (*n* = 10), according to their initial shrunk BW (after 14 h of feed and water restriction; initial BW = 212 ± 3.1 kg and age = 16 ± 3.0 mo). The experimental period lasted 140 d, divided into five periods of 28 d each. Bulls were kept in a covered feedlot (10 pens per treatment; 4 bulls per pen; 3 × 6 m) with a concrete floor, feed and mineral bunk, and waterer. Within blocks (*n* = 10), animals were randomly assigned to the same treatments as in Exp. 1.

Bulls were fed daily, and diets (Table 1) were composed of 96% of chopped coastcross haylage [*C. dactylon* (L.) Pers] and 4% of concentrate (composed of 50% ground corn and 50% ground citrus pulp dry), used as a delivery vehicle for the additives. Haylage was chopped using a vertical mixer (Mixer VM8B; DeLaval International AB, Tumba, Sweden) for about 15 min, which resulted in an average particle length distribution of 60.1% ± 1.8% > 19 mm; 23.0% ± 1.0% > 8 mm; 8.9% ± 1.7% > 4 mm; and 8.0% ± 1.3% on bottom sieve according to Penn State Particle Separator procedures (Heinrichs, 1996; Kononoff et al., 2003). Feed additives were mixed into the concentrate and offered to each pen individually. Bulls promptly consumed the concentrate within 20 min after feeding, and then haylage was offered. Treatments were provided daily before haylage feeding to avoid the small amount of concentrate mixed with the haylage and compromise the intake of feed additives. Bulls were fed the treatments (concentrate with or without feed additives) once daily at 0730 h and had ad libitum access to haylage (0800 h), mineral–vitamin mix, and fresh water. Mineral mix (Bellmais; Trouw Nutrition) used herein was the same as in Exp. 1 and was offered separately in feed bunk from haylage and treatments. The initial inclusion of additives in the concentrate was also the same as in Exp. 1 based on 5 kg of forage DMI and additives doses were according to the manufacturer’s recommendation. Throughout the experimental period (day 0 to 140), the additives dosage offered to the animals was based on the total DMI of the previous day.

#### Sample collection, laboratory analyses, and measurements

To calculate average daily gain (**ADG**) and gain:feed (**G:F**) efficiency, bulls were individually weighed on day 0, 28, 56, 84, 112, and 140 (final days of each period) after 14 h of feed and water restriction. DMI was evaluated daily from each pen within each period by collecting and weighing nonconsumed haylage weekly. Haylage and total DMI of each pen were divided by the number of bulls within each pen and expressed as kg per bull/day. Total BW gain and DMI of each period were used for bull G:F calculation. Samples of feed and orts were collected weekly, pooled across all weeks within each period, and analyzed for nutrient profile as aforementioned for Exp. 1.

### Statistical analysis

Data were analyzed using animal (Exp. 1) or pen (Exp. 2) as the experimental unit and the MIXED procedure of SAS (SAS Institute Inc., Cary, NC), with Satterthwaite approximation to determine the denominator df for the test of fixed effects, and animal(treatment) as random variable for Exp. 1. In Exp. 2, however, pen(treatment) and animal(pen × treatment) were used as random variables for all variables, except for DMI and G:F that used pen(treatment) as random variables. Model statements contained the effects of treatment, period or day, treatment × day or period interaction, and block as an independent variable. The specified term for all repeated statements was day or period, with animal(treatment) as a subject for Exp. 1, whereas in Exp. 2, pen(treatment) was used as a subject for DMI and G:F only, and animal(pen × treatment) as a subject for all other analyses following the rationale described by St-Pierre (2007) and Bello et al. (2016). The covariance structure was first-order autoregressive, which provided the best fit for these analyses according to the smallest Akaike Information Criterion. Results from Exp. 1 are reported as covariately adjusted least square means for values obtained on day 0, except for forage DMI, and separated using PDIFF. All results from Exp. 2 are reported as least square means and were separated using PDIFF. Significance was set at *P* ≤ 0.05, and tendencies were determined if *P* > 0.05 and ≤0.10. Results are reported according to the main effects if no interactions were significant.

## Results

### Experiment 1—Rumen-cannulated steers

**Figure 1.**
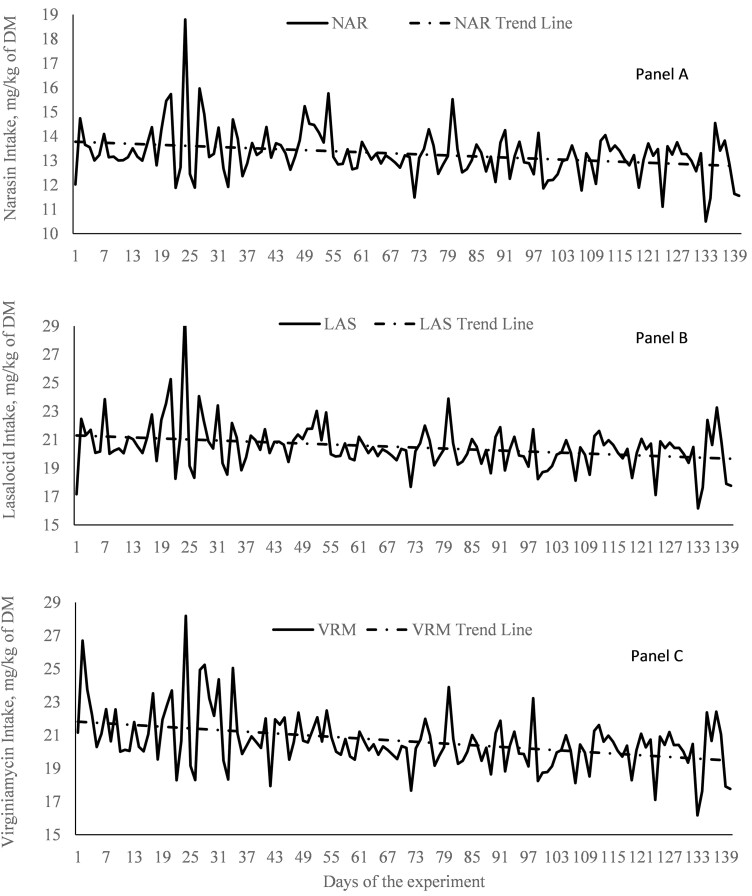
Feed additives intake of rumen-cannulated *Bos indicus* Nellore steers receiving forage-based diets supplemented or not (CON, *n* = 8) with 13 mg/kg of dry matter (DM) of narasin (NAR, *n* = 8; Zimprova; Elanco Animal Health, São Paulo, SP, Brazil; Panel A), 20 mg/kg of DM of lasalocid (LAS; *n* = 8; Taurotec, Zoetis, Sao Paulo, SP, Brazil; Panel B), or 20 mg/kg of DM of virginiamycin (VRM; *n* = 8; V-Max, Phibro Animal Health Corporation, Guarulhos, SP, Brazil; Panel C) for 140 days (Exp. 1).

The average daily intake of feed additive was 13.3 ± 0.08, 20.4 ± 0.14, and 20.6 ± 0.15 mg/kg of DM per day for NAR, LAS, and VRM, respectively (Figure 1), which followed the manufacturer’s recommendation and previous day forage DMI. SCFA values obtained on day 0 of the study were not significant covariates (*P* > 0.68) for molar proportions of acetate, propionate, isobutyrate, butyrate, isovalerate, and valerate, and did not differ between treatments (*P* > 0.37; data not shown), indicating that animals had similar dietary management before the beginning of the present study.

No treatment × period interactions were detected (*P* ≥ 0.32) for intake and apparent digestibility of nutrients among treatments (Table 2). In contrast, day effects were detected (*P* < 0.01) for these variables (Table 2), which was expected due to daily haylage quality variation. Hence, the inclusion of dietary additives did not affect (main treatment effect; *P* > 0.31) intake and apparent digestibility of nutrients.

**Table 2.** Intake and total tract apparent digestibility of nutrients in rumen-cannulated *Bos indicus* Nellore steers receiving a forage-based diets supplemented or not (CON, *n* = 8) with narasin (NAR, *n* = 8), lasalocid (LAS; *n* = 8), or virginiamycin (VRM; *n* = 8) for 140 days (Exp. 1)

	Treatments^1^					*P*-value^2^		
Item	CON	NAR	LAS	VRM	SEM	Treatment	Period	T × P
Intake, kg/d								
Dry matter	6.50	6.99	6.61	6.62	1.28	0.46	<0.01	0.65
Organic matter	5.77	6.37	5.95	5.97	1.27	0.33	<0.01	0.32
Neutral detergent fiber	4.17	4.49	4.24	4.27	0.82	0.43	<0.01	0.57
Acid detergent fiber	2.20	2.42	2.24	2.25	0.48	0.31	<0.01	0.71
Crude protein	0.75	0.82	0.77	0.77	0.14	0.33	<0.01	0.49
Digestibility, % (dry matter basis)								
Dry matter	54.94	57.29	55.76	55.35	1.12	0.48	<0.01	0.36
Organic matter	56.99	59.21	57.84	57.48	1.17	0.36	<0.01	0.88
Neutral detergent fiber	61.49	64.18	62.67	61.79	1.15	0.34	<0.01	0.73
Acid detergent fiber	60.84	62.41	60.93	60.48	1.29	0.72	<0.01	0.51
Crude protein	55.79	57.94	57.71	57.87	1.16	0.48	<0.01	0.82

^1^CON, no feed additives; NAR, inclusion of 13 mg/kg of dry matter of narasin (Zimprova; Elanco Animal Health, São Paulo, SP, Brazil); LAS, inclusion of 20 mg/kg of dry matter of lasalocid (Taurotec; Zoetis, Sao Paulo, SP, Brazil; *n* = 8); VRM, inclusion of 20 mg/kg of dry matter of virginiamycin (V-Max; Phibro Animal Health Corporation, Guarulhos, SP, Brazil; *n* = 8).

^2^ *P*-value for treatment, period, and treatment × period interaction (T × P).

**Figure 2.**
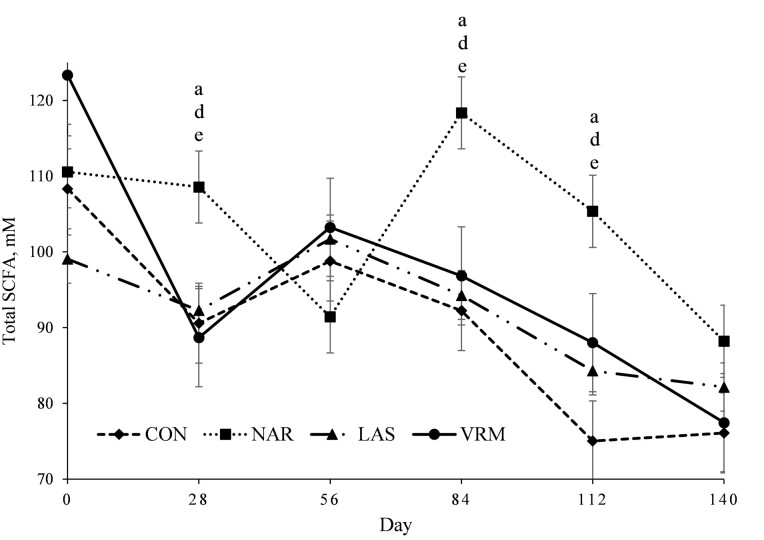
Molar proportion of total short-chain fatty acids (SCFA) of rumen-cannulated *Bos indicus* Nellore steers receiving forage-based diets supplemented or not (CON, *n* = 8) with 13 mg/kg of dry matter (DM) of narasin (NAR, *n* = 8; Zimprova; Elanco Animal Health, São Paulo, SP, Brazil), 20 mg/kg of DM of lasalocid (LAS; *n* = 8; Taurotec; Zoetis, Sao Paulo, SP, Brazil), or 20 mg/kg of DM of virginiamycin (VRM; *n* = 8; V-Max; Phibro Animal Health Corporation, Guarulhos, SP, Brazil) for 140 days (Exp. 1). Treatments were offered daily throughout the experimental period (day 0 to 140). Rumen samples were collected on day 0 (prior to first treatment administration), 28, 56, 84, 112, and 140 of the study. Data were analyzed using results from day 0 as an independent covariate. Within days, letters indicate treatment comparisons (*P* ≤ 0.05): a = CON vs. NAR, b = CON vs. LAS, c = CON vs. VRM, d = NAR vs. LAS, e = NAR vs. VRM, and f = LAS vs. VRM.

**Figure 3.**
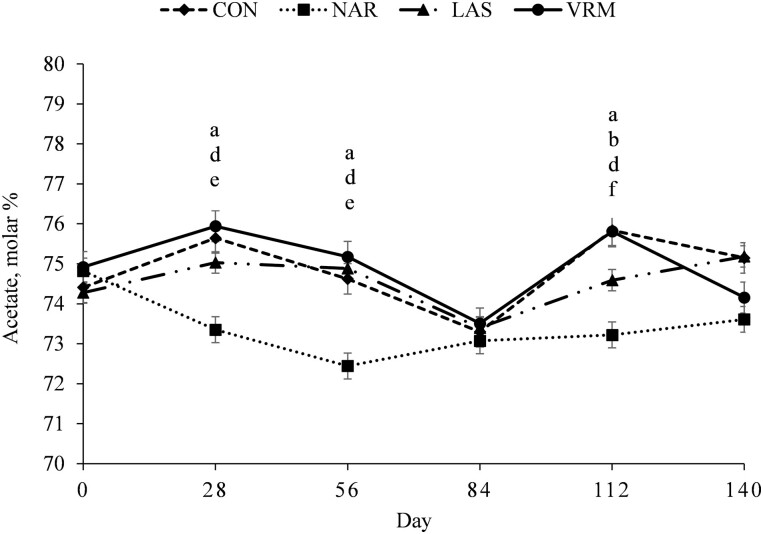
Molar proportion of acetate of rumen-cannulated *Bos indicus* Nellore steers receiving forage-based diets supplemented or not (CON, *n* = 8) with 13 mg/kg of dry matter (DM) of narasin (NAR, *n* = 8; Zimprova; Elanco Animal Health, São Paulo, SP, Brazil), 20 mg/kg of DM of lasalocid (LAS; *n* = 8; Taurotec; Zoetis, Sao Paulo, SP, Brazil), or 20 mg/kg of DM of virginiamycin (VRM; *n* = 8; V-Max; Phibro Animal Health Corporation, Guarulhos, SP, Brazil) for 140 days (Exp. 1). Treatments were offered daily throughout the experimental period (day 0 to 140). Rumen samples were collected on day 0 (prior to first treatment administration), 28, 56, 84, 112, and 140 of the study. Data were analyzed using results from day 0 as an independent covariate. Within days, letters indicate treatment comparisons (*P* ≤ 0.05): a = CON vs. NAR, b = CON vs. LAS, c = CON vs. VRM, d = NAR vs. LAS, e = NAR vs. VRM, and f = LAS vs. VRM.

**Figure 4.**
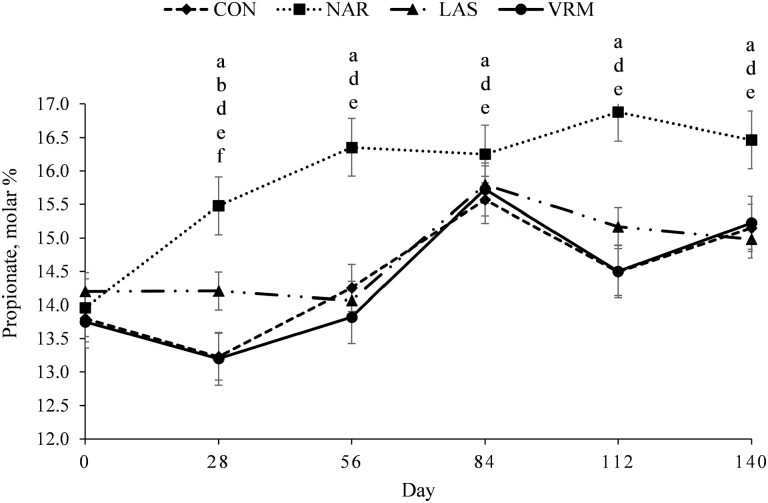
Molar proportion of propionate of rumen-cannulated *Bos indicus* Nellore steers receiving forage-based diets supplemented or not (CON, *n* = 8) with 13 mg/kg of dry matter (DM) of narasin (NAR, *n* = 8; Zimprova; Elanco Animal Health, São Paulo, SP, Brazil), 20 mg/kg of DM of lasalocid (LAS; *n* = 8; Taurotec; Zoetis, Sao Paulo, SP, Brazil), or 20 mg/kg of DM of virginiamycin (VRM; *n* = 8; V-Max; Phibro Animal Health Corporation, Guarulhos, SP, Brazil) for 140 days (Exp. 1). Treatments were offered daily throughout the experimental period (day 0 to 140). Rumen samples were collected on day 0 (prior to first treatment administration), 28, 56, 84, 112, and 140 of the study. Data were analyzed using results from day 0 as an independent covariate. Within days, letters indicate treatment comparisons (*P* ≤ 0.05): a = CON vs. NAR, b = CON vs. LAS, c = CON vs. VRM, d = NAR vs. LAS, e = NAR vs. VRM, and f = LAS vs. VRM.

**Figure 5.**
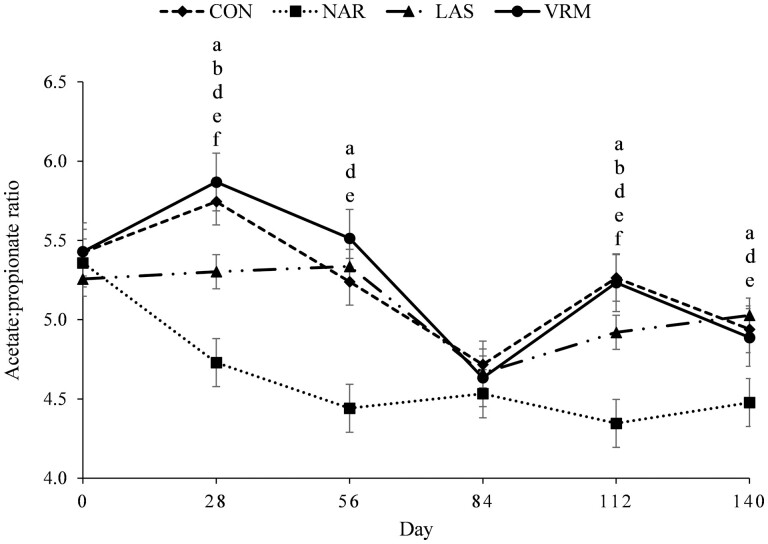
Molar proportion of acetate:propionate ratio of rumen-cannulated *Bos indicus* Nellore steers receiving forage-based diets supplemented or not (CON, *n* = 8) with 13 mg/kg of dry matter (DM) of narasin (NAR, *n* = 8; Zimprova; Elanco Animal Health, São Paulo, SP, Brazil), 20 mg/kg of DM of lasalocid (LAS; *n* = 8; Taurotec; Zoetis, Sao Paulo, SP, Brazil), or 20 mg/kg of DM of virginiamycin (VRM; *n* = 8; V-Max; Phibro Animal Health Corporation, Guarulhos, SP, Brazil) for 140 days (Exp. 1). Treatments were offered daily throughout the experimental period (day 0 to 140). Rumen samples were collected on day 0 (prior to first treatment administration), 28, 56, 84, 112, and 140 of the study. Data were analyzed using results from day 0 as an independent covariate. Within days, letters indicate treatment comparisons (*P* ≤ 0.05): a = CON vs. NAR, b = CON vs. LAS, c = CON vs. VRM, d = NAR vs. LAS, e = NAR vs. VRM, and f = LAS vs. VRM.

A treatment × day interaction was noted (*P* ≤ 0.03) for total ruminal SCFA (Figure 2), acetate (Figure 3), propionate (Figure 4), and Ac:Pr ratio (Figure 5). Steers receiving NAR had greater (*P* < 0.01) total ruminal SCFA values on day 28, 84, and 112 of the study when compared with LAS, VRM, and CON steers, whereas total ruminal SCFA did not differ (*P >* 0.51) between LAS, VRM, and CON steers (Table 3). Steers fed NAR had the lowest (*P* < 0.01) molar proportion of acetate on day 28, 56, and 112 compared with CON, LAS, and VRM steers, whereas it did not differ (*P* > 0.25) between LAS, VRM, and CON steers from day 28 to 84. On day 112, steers fed LAS had a lower (*P* < 0.02) molar proportion of acetate compared with VRM and CON steers, whereas no differences were noted between CON and VRM steers (*P* > 0.33). Contrary, steers receiving NAR had a greater (*P* < 0.04) molar proportion of ruminal propionate during the entire experimental period, except on day 84 (*P* > 0.21), compared with CON, LAS, and VRM steers, whereas LAS steers had greater (*P* < 0.04) ruminal propionate compared with CON and VRM steers on day 28 and 112, and it did not differ (*P* > 0.22) between CON and VRM throughout the experimental period. Consequently, NAR steers had the lowest (*P* < 0.04) Ac:Pr ratio during the entire experimental period, except day 84 (*P* > 0.16), whereas LAS steers had a lower (*P* < 0.04) ratio compared with CON and VRM steers on day 28 and 112, and it did not differ (*P* > 0.34) between CON and VRM throughout the experimental period. No treatment effects were detected (*P* ≥ 0.27) on the molar proportion of ruminal butyrate, isobutyrate, valerate and isovalerate (Table 3).

**Table 3.** Molar proportion of ruminal short-chain fatty acids (SCFA), ammonia, and ruminal pH of rumen-cannulated *Bos indicus* Nellore steers receiving forage-based diets supplemented or not (CON, *n* = 8) with narasin (NAR, *n* = 8), lasalocid (LAS; *n *= 8), or virginiamycin (VRM; *n *= 8) for 140 days (Exp. 1)

	Treatments^1^					*P-*value^2^		
Item	CON	NAR	LAS	VRM	SEM	Treatment	Day	T × D
Rumen pH	6.85^b^	6.79^b^	6.84^b^	6.98^a^	0.04	0.03	<0.01	0.19
Ammonia, mg/dL	4.18	4.28	4.25	4.87	0.77	0.11	<0.01	0.17
Total SCFA, mM	87.13^b^	101.51^a^	87.17^b^	90.35^b^	6.92	0.02	<0.01	<0.01
SCFA, mM/100mM^3^								
Acetate	74.91^a^	73.14^b^	74.61^a^	74.98^a^	0.38	<0.01	<0.01	<0.01
Propionate	14.53^b^	16.28^a^	14.84^b^	14.49^b^	0.22	<0.01	<0.01	<0.01
Butyrate	7.80	7.75	7.62	7.71	0.14	0.78	<0.01	0.54
Isobutyrate	0.87	0.87	0.88	0.89	0.02	0.84	<0.01	0.47
Valerate	0.87	0.85	0.85	0.88	0.08	0.95	<0.01	0.87
Isovalerate	1.05	1.10	1.11	1.09	0.02	0.27	<0.01	0.11
Acetate:propionate	5.18^b^	4.52^a^	5.04^b^	5.20^b^	0.06	<0.01	<0.01	<0.01

^1^CON, no feed additives; NAR, inclusion of 13 mg/kg of dry matter (DM) of narasin (Zimprova; Elanco Animal Health, São Paulo, SP, Brazil); LAS, inclusion of 20 mg/kg of DM of lasalocid (Taurotec; Zoetis, Sao Paulo, SP, Brazil; *n* = 8); VRM, inclusion of 20 mg/kg of DM of virginiamycin (V-Max; Phibro Animal Health Corporation, Guarulhos, SP, Brazil; *n* = 8).

^2^ *P-*value for treatment, day, and treatment × day interaction (T × D).

^3^On day 0 (immediately prior to the beginning of the experimental period and first treatment offer), 28, 56, 84, 112, and 140 of the experimental periods at 0, 6, and 12 h after feeding concentrate + treatments, ruminal fluid samples were collected (approximately 100 mL).

Steers receiving VRM had greater (main treatment effect; *P* = 0.03) rumen pH compared with CON, LAS, and NAR steers, whereas it did not differ (*P* > 0.84) between CON, LAS, and NAR (Table 3). No treatment differences (*P* ≥ 0.11) were detected for rumen ammonia (Table 3), and plasma glucose and urea concentration (Table 4), but a day effect was detected (*P* < 0.01) for these variables.

**Table 4.** Plasma glucose and urea concentration of rumen-cannulated *Bos indicus* Nellore steers receiving forage-based diets supplemented or not (CON, *n* = 8) with narasin (NAR, *n* = 8), lasalocid (LAS; *n* = 8), or virginiamycin (VRM; *n* = 8) for 140 days (Exp. 1)

	Treatments^1^					*P-*value^2^		
Item^3^	CON	NAR	LAS	VRM	SEM	Treatment	Day	T × D
Glucose, mg/dL	69.37	69.30	68.63	70.53	1.52	0.76	<0.01	0.51
Urea, mg/dL	32.46	33.74	32.11	34.56	1.36	0.53	<0.01	0.60

^1^CON, no feed additives; NAR, inclusion of 13 mg/kg of dry matter (DM) of narasin (Zimprova; Elanco Animal Health, São Paulo, SP, Brazil); LAS, inclusion of 20 mg/kg of DM of lasalocid (Taurotec; Zoetis, Sao Paulo, SP, Brazil; *n* = 8); VRM, inclusion of 20 mg/kg of DM of virginiamycin (V-Max; Phibro Animal Health Corporation, Guarulhos, SP, Brazil; *n* = 8). Within rows, values with different superscripts differ (*P* ≤ 0.05).

^2^ *P-*value for treatment, day, and treatment × day interaction (T × D).

^3^Blood samples were collected on day 0 (immediately prior to the beginning of the experimental period and first treatment offer), 28, 56, 84, 112, and 140 of the experimental period and 6 h after feeding, via coccygeal venipuncture into commercial vacutainer collection tubes with glycolytic inhibitor and anticoagulant K3EDTA (Vacuette; Greiner Bio-One, Americana, SP, Brazil).

### Experiment 2—Animal performance

Based on manufacturer’s recommendation and the previous day forage DMI, the feed additives consumption during Exp. 2 was of 14.7 ± 0.8, 21.1 ± 0.8, and 21.4 ± 0.8 mg/kg of DM of for NAR, LAS, and VRM respectively. As expected and based on the experimental design, initial BW was similar among treatments (*P* = 0.61; Table 5).

**Table 5.** Performance parameters of *Bos indicus* Nellore yearling Bulls receiving forage-based diets supplemented or not (CON, *n* = 10) with narasin (NAR, *n* = 10), lasalocid (LAS; *n* = 10), or virginiamycin (VRM; *n* = 10) for 140 days (Exp. 2)

	Treatments^2^					*P-*value^3^		
Item^1^	CON	NAR	LAS	VRM	SEM	Treatment	Day	T × D
Body weight, kg								
Initial (day 0)	214	211	210	215	3.1	0.61	—	—
Day 28	228.7	232.6	232.5	230.6	1.9	0.43	—	—
Day 56	244.2	251.1	248.2	245.5	2.2	0.14	—	—
Day 84	255.0^b^	264.3^a^	258.0^ab^	254.8^b^	2.8	0.09	—	—
Day 112	262.4^b^	275.5^a^	264.8^b^	263.7^b^	3.2	0.03	—	—
Final (day 140)	273.9^b^	287.8^a^	277.1^b^	275.7^b^	3.4	0.03	—	—
Dry matter intake, kg	5.26^b^	5.69^a^	5.16^b^	5.11^b^	0.14	0.03	<0.01	0.99
Average daily gain, kg	0.451^b^	0.557^a^	0.498^ab^	0.459^b^	0.03	0.04	<0.01	0.16
Feed efficiency, g/kg	80.3^c^	95.2^a^	92.1^ab^	84.6^bc^	4.4	0.09	<0.01	0.40

^1^On day 0 of the experimental period, individual shrunk body weight (BW) was recorded after 14 h of feed and water withdrawal to determine animal initial BW. To calculate average daily gain and feed efficiency, bulls were individually weighed on days 0, 28, 56, 84, 112, and 140 (final days of each period) after 14 h of feed and water restriction. Dry matter intake (DMI) was evaluated daily from each pen within each period by collecting and weighing nonconsumed feed weekly. Hay and total DMI of each pen were divided by the number of bulls within each pen and expressed as kg per bull/day. Total BW gain and DMI of each period were used for bull feed efficiency calculation.

^2^CON, no feed additives; NAR, inclusion of 13 mg/kg of dry matter (DM) of narasin (Zimprova; Elanco Animal Health, São Paulo, SP, Brazil); LAS, inclusion of 20 mg/kg of DM of lasalocid (Taurotec; Zoetis, Sao Paulo, SP, Brazil; *n* = 8); VRM, inclusion of 20 mg/kg of DM of virginiamycin (V-Max; Phibro Animal Health Corporation, Guarulhos, SP, Brazil; *n* = 8). Within rows, values with different superscripts differ (*P* ≤ 0.05).

^3^ *P-*value for treatment, day, and treatment × day interaction (T × D).

Nellore bulls fed NAR had greater (main treatment effect; *P* < 0.02) ADG compared with CON and VRM bulls, and similar (*P* = 0.17) ADG between NAR and LAS bulls, whereas ADG did not differ (*P* > 0.28) between LAS, VRM, and CON bulls (Table 5). A treatment effect was detected (*P* = 0.03) for DMI, being greater in NAR bulls compared with CON, LAS, and VRM bulls, and similar (*P* > 0.48) between CON, LAS, and VRM bulls. A tendency was detected (main treatment effect; *P* = 0.09) for G:F, which was greater (*P* < 0.02) in NAR bulls compared with CON and VRM, and similar (*P* = 0.36) between NAR and LAS bulls. However, G:F was also greater (*P* = 0.05) in bulls receiving LAS vs. CON, it did not differ (*P* = 0.24) between LAS and VRM, and remained similar (*P* = 0.49) between CON and VRM bulls. Beginning on day 84, a ­tendency was detected (*P* = 0.09) for BW, which was greater (*P* < 0.02) in bulls receiving NAR compared with CON and VRM, similar (*P* > 0.13) between NAR and LAS bulls, and it did not differ (*P* > 0.43) between CON, LAS, and VRM bulls. From day 112 to 140, bulls receiving NAR had greater (*P* < 0.03) BW compared with CON, LAS, and VRM bulls, whereas BW on these days did not differ (*P* > 0.51) between CON, LAS, and VRM bulls.

## Discussion

Beef cattle production systems in tropical and temperate regions typically rely on forage-based diets for extended periods during the annual production cycle. However, seasonal variations in forage quality and quantity often affect nutrient utilization and performance of beef cattle by limiting energy and protein intake (Hills et al., 2015; de Souza et al., 2017). Feed additives are an essential nutritional management tool used to enhance productivity and profitability of beef ­cattle production systems by changing the microbial ecosystem and fermentation dynamics in the rumen, along with effective nutrient utilization and energy of the diet (Tedeschi et al., 2003; Weimer et al., 2008; Schären et al., 2017; Marques and Cooke, 2021). Nevertheless, there are several ionophores (LAS, monensin, salinomycin, laidlomycin, and NAR) and nonionophores (VRM and bambermycin) commercially available for animal consumption that have similar effects on animal performance, but their mechanisms in the rumen may vary depending on dosage, animal, frequency of use, and diet (Nagaraja et al., 1987; Tedeschi et al., 2003; Bretschneider et al., 2008; Limede et al., 2021; Soares et al., 2021). Furthermore, there is limited or inconsistent evidence about the impacts of feed additives on intake, ruminal fermentative parameters, and performance of Nellore cattle fed forage-based diet (Bretschneider et al., 2008).

In the present study, only animals fed NAR had an overall ADG of 23% and 21% greater than the inclusion or not (CON) of VRM in forage-based diets, respectively. These outcomes partially resulted from the differences in DMI and ruminal fermentation profile in animals fed NAR. In fact, NAR supplementation in forage-based diets increased DMI by 8.1%, 10.2%, and 11.3% compared with bulls supplemented with CON, LAS, and VRM, respectively. These increments in ADG and DMI for NAR benefited bulls G:F by 18.5% and 12.5% when compared with bulls fed CON and VRM, respectively. Nevertheless, NAR supplementation was the only feed additive that improved final BW by 13.9, 10.8, and 12.1 kg compared with CON, LAS, and VRM bulls fed forage-based diets, respectively. It is well known that ionophores and nonionophores influence ADG, DMI, and G:F of animals fed a high-concentrate diet (Rogers et al., 1995; Duffield et al., 2012; Golder and Lean, 2016; Tedeschi and Gorocica-Buenfil, 2018). When fed to grazing animals, ionophores and nonionophores also enhanced performance parameters, except for DMI, which might depend on forage characteristics, passage rate, and gut fill of the animals (Bretschneider et al., 2008; Ellis et al., 2008). Accordingly, others have also shown that the inclusion of NAR (Silva et al., 2015; Pasqualino et al., 2020; Polizel et al., 2020), LAS, monensin, or flavomycin (DelCurto et al., 1998; Limede et al., 2021) did not influence DMI in animals fed forage-based diets. Conversely, and supporting our results, Polizel et al. (2021) reported that supplementing NAR to lambs consuming low- (7% CP) or medium- (11.7% CP) quality forage increased DMI, ­regardless of forage quality. The interaction between forage quality and additive doses can drive the DMI in livestock animals in forage-based systems (Bretschneider et al., 2008; Ellis et al., 2012). Accordingly, Limede et al. (2021) reported greater DMI and ADG in Nellore bulls fed NAR, resulting in heavier animals at the end of the supplementation period compared with the addition or not of ionophores and nonionophores in high-quality forage-based diets (approximately 18% CP). The data obtained in the present study demonstrate that NAR supplementation also increases the DMI of growing steers fed medium-quality forage (12% CP), indicating a potential use of the molecule in different conditions of forage-based systems.

The increments in performance parameters of Nellore bulls fed NAR might also be explained by the differences in ruminal SCFA profile, given that altering ruminal fermentation toward propionate and decreasing acetate concentration are positively correlated with greater feed energy utilization and performance (Blaxter, 1962; Russel and Strobel, 1989; McGuffey et al., 2001; Weimer et al., 2008). Specifically, NAR supplementation improved the molar concentration of propionate by 12.0%, 9.7%, and 12.3%, decreased acetate by 2.3%, 1.9%, and 2.4%, and improved total SCFA by 16.5%, 16.4%, 12.3% compared with bulls supplemented with CON, LAS, and VRM, respectively. Accordingly, others have also shown increased concentrations of ruminal propionate and total SCFA, and reduced concentration of rumen acetate when NAR was supplemented in forage-based diets (Polizel et al., 2020; Limede et al., 2021). These results may also be explained by the fact that NAR has similar ruminal mechanisms as other ionophores. Ionophores alter rumen microbial population through ion transfer across cell membranes (Berg and Hamill, 1978), resulting in enhanced efficiency of energy and nitrogen metabolism of rumen bacteria and/or animals (Russell and Strobel, 1989; McGuffey et al., 2001; Guan et al., 2006), which translates into improved performance in beef cattle (Duffield et al., 2012; Limede et al., 2021). Contrariwise, LAS, another ionophore used in the present study, was not capable of altering ruminal fermentation parameters, ADG, and final BW in beef cattle fed forage-based diets. These results are not in accordance with the meta-analysis reported by Golder and Lean (2016), in which LAS supplementation improved the concentration of SCFA and propionate, and ADG and feed efficiency compared with nonsupplemented animals. Nevertheless, sorting studies only by forage-based diets (five studies), four studies have shown an increase in total SCFA, whereas only one study increased propionate (Golder and Lean, 2016). The lack of response in ruminal fermentation parameters and performance of animals fed forage-based diets in the meta-analysis by Golder and Lean (2016) and our study might be explained by the LAS dose. The LAS dose used herein was 20 mg/kg DM (approximately 132 mg/d in Exp. 1 and 103 mg/d in Exp. 2), similar to the doses used in metabolism and performance trials reported by the meta-analysis. Accordingly, several studies reported no benefits of supplementing LAS to forage-based diets on animal performance and rumen fermentation parameters when the dose is below 200 mg/day (Andersen and Horn, 1987; Goetsch et al., 1993). Hence, we could assume that the dose used herein was not appropriate to detect differences in rumen fermentation and performance parameters in cattle fed forage-based diets, given that we follow the manufacturer’s recommendation and ionophores have dose-dependent effects (Bretschneider et al., 2008; Ellis et al., 2012).

VRM is a nonionophore molecule active against gram-positive bacteria by impairing its protein synthesis, resulting in the inhibition of multiplication and, eventually, cellular death (Cocito, 1979). The primary effect of VRM is on lactic acid concentration in the rumen when ruminants are fed a high-concentrate diet, reducing the risk of rumen acidosis and hepatic abscesses, which might benefit animal performance and health (Rogers et al., 1995; Tedeschi and Gorocica-Buenfil, 2018). However, the effects of VRM supplementation to beef cattle fed forage-based diets have been inconsistent on ruminal fermentation and performance parameters (Fiems et al., 1992; Ferreira et al., 2015; Maciel et al., 2019). For instance, Maciel et al. (2019) reported that supplementing 84 to 100 mg/day of VRM did not impact performance or intake of grazing Nellore heifers. Salinas-Chavira et al. (2009) stated that supplementation of Holstein steers with 22.5 mg of VRM/kg of DMI did not impact ruminal fermentation parameters and performance, corroborating the results obtained in this study.

It is well-established that rumen ammonia concentration below 5 mg/dL may limit microbial growth and ruminal fermentation parameters (Satter and Slyter, 1974; Slyter et al., 1979). Moreover, dietary feed additives might reduce proteolysis and amino acid deamination and, consequently, ruminal ammonia production (Goodrich et al., 1984; Rogers et al., 1995), resulting in diminished absorption and blood urea concentration (Russell and Martin, 1984; Chen and Russell, 1991). Still, the ionophores and nonionophores used herein did not impact ruminal ammonia and blood urea concentration of beef steers fed a forage-based diet, even with the differences observed in the ruminal SCFA profile. Research from our group recently reported that supplementing ionophores or nonionophores to beef cattle receiving a forage-based diet also did not impact ruminal ammonia concentration (Limede et al., 2021). Accordingly, Lemos et al. (2016) and Bell et al. (2017) reported no difference in ruminal ammonia concentration of beef cattle receiving ionophores or nonionophores in forage- or concentrate-based diets.

The impacts of feed additives on apparent nutrient digestibility are still uncertain in the literature (Wedegaertner and Johnson, 1983; Ricke et al., 1984; Crossland et al., 2017; Polizel et al., 2020; Limede et al., 2021). Adding ionophores or nonionophores into forage-based diets did not affect apparent nutrient digestibility in the current study (Exp. 1), which is in agreement with several studies from others (Reffett-Stabel et al., 1989; DelCurto et al., 1998; Bell et al., 2017) and our research group (Polizel et al., 2020; Limede et al., 2021). Therefore, comparing our findings with those of other studies suggests that the main effect of feed additives is on the rumen environment, altering the ruminal stoichiometry by changing SCFA production and increasing dietary energy utilization.

Generally, ruminal fiber digestion is depressed when the ruminal pH declines below 6.2 (Grant and Mertens, 1992). In the present study, all animals consumed forage-based diets, and only a small amount of concentrate was used to deliver the treatments. Hence, ruminal pH values in all treatments were in a range that would not impair ruminal digestibility or bacteria function (Bell et al., 2017; Crossland et al., 2017). Still, in this study, animals receiving VRM had greater ruminal pH values than cohorts, which might be partially explained by the effects of VRM on lactic acid-producing bacteria, limiting ruminal lactate accumulation (Nagaraja et al., 1987; Salinas-Chavira et al., 2009). Research from others (Cushman et al., 2009; Bell et al., 2017) and our group (Pasqualino et al., 2020; Polizel et al., 2020; Limede et al., 2021) also demonstrated that ruminal pH of beef steers offered a forage-based diet was not impacted by the addition of ionophores or nonionophores in diet, within a range that provides adequate forage digestibility (Yokoyama and Johnson, 1988).

Improving ruminal fermentation parameters toward propionate production might increase glucose concentration through hepatic gluconeogenic flux (Duffield et al., 2008). Although NAR supplementation was the only additive that increased ruminal propionate production herein, serum glucose concentration was not altered. In agreement with these results, Bohnert et al. (2016) reported that monensin supplementation also impacted ruminal propionate concentration, whereas it did not change the glucose concentrations of ruminants consuming low-quality forage. Polizel et al. (2020) also reported an increase in ruminal propionate with no effect in plasma glucose concentration of steers supplemented with NAR into a forage-based diets.

In summary, the inclusion of ionophores and nonionophores in forage-based diets did not affect nutrient intake and apparent digestibility of nutrients (Exp. 1). Nonetheless, ruminal fermentation profile (Exp. 1) and intake (Exp. 2) were impacted by the addition of NAR into forage-based diets of Nellore cattle. These results might have contributed partially to the enhanced performance of Nellore bulls receiving NAR compared with CON and VRM cohorts when a forage-based diet was offered. The lack of effects on ruminal fermentation characteristics, ADG, and final BW for LAS and VRM could be attributed to the dose used in the current experiment, despite the manufacture’s recommendation. Nevertheless, this research provides insights into NAR as an important feed additive for forage-based beef cattle diets. Further research should be undertaken to investigate the appropriate dosage of ionophores and nonionophores when used in forage-based beef cattle systems.

## Abbreviations

Ac:Pr: acetate:propionate
ADF: acid detergent fiber
ADG: average daily gain
BW: body weight
CON: forage-based diet without feed additives
CP: crude protein
DM: dry matter
DMI: dry matter intake
FDM: fecal dry matter
G:F: feed efficiency
LAS: lasalocid
NAR: narasin
NCDM: nutrient content of the dry matter intake
NCFM: nutrient content of the fecal dry matter
NDF: neutral detergent fiber
SCFA: short-chain fatty acids
TTAD: total tract apparent digestibility
VRM: virginiamycin
